# A thermodynamic insight into viral infections: do viruses in a lytic cycle hijack cell metabolism due to their low Gibbs energy?

**DOI:** 10.1016/j.heliyon.2020.e03933

**Published:** 2020-05-08

**Authors:** Marko Popovic, Mirjana Minceva

**Affiliations:** Biothermodynamics, TUM School of Life Sciences Weihenstephan, Technical University of Munich, Maximus-von-Imhof-Forum 2, 85354, Freising, Germany

**Keywords:** Microbiology, Biophysics, Thermodynamics, Chemical reaction kinetics, Biophysical chemistry, Virology, Virus, Organism empirical formula, Growth reaction, Gibbs free energy of growth, Growth rate

## Abstract

After adsorption and penetration, a virus hijacks a cell's metabolic machinery and uses it as a medium for its reproduction and growth through multiplication. Growth is competitive, since the same precursors and machinery are used by both the virus and its host cell. But what drives a virus to perform its life cycle more efficiently than its host? Gibbs energy represents the driving force for all chemical reactions in nature. Therefore, hypothetically Gibbs energy of growth can represent the driving force of viral lytic cycle. After chemical characterization of 17 viruses and their hosts, in this paper, growth reactions were suggested, and enthalpy, entropy and Gibbs free energy of both formation and growth were calculated. By comparing the Gibbs energy of growth of viruses and their hosts, it has been found that a virus always has a more negative Gibbs free energy of growth than its host implying that synthesis of viral components is more thermodynamically favorable. Thus, it seems that the physical laws explain observed biological phenomena - the hijack of host life machinery and high efficiency of virus growth.

## 1 Introduction

During the golden age of virology, several thousand viruses have been discovered, morphologically characterized and classified [Norrby, 2008]. Viruses are composed of a nucleic acid, bordered by a protein capsid and sometimes a lipid envelope as well. A real or imagined amount of substance, separated from its environment represents a thermodynamic system. Thus, a virus nucleic acid bordered by a capsid represents an open (bio)thermodynamic system [Von Bertalanffy, 1950]. It has been observed that a virus performs a life cycle, hijacking its host cell's vital machinery, performing replication, transcription, translation and self-assembly processes. However, a question remains open: what drives a virus to perform its life cycle more efficiently than its host? If the driving force of viral life cycle were known, it would be possible to influence viral multiplication rate and thereby their virulence.

The driving force of all processes in nature, including growth of organisms is a thermodynamic property – Gibbs energy [Demirel, 2014; Von Stockar, 2014]. Thermodynamics has played a fundamental role in development of science, allowing us to understand a wide range of natural phenomena and design new technologies. The potential of thermodynamics in life sciences was first noticed by Boltzmann [1974], whose ideas were extended by Schrödinger [1944]. However, its use in life sciences and bioengineering is hindered by lack of data for thermodynamic analysis [Von Stockar, 2010]. Thus, to fundamentally understand life and interactions between organisms (e.g. parasitism, infections, immune answer etc.), we need to chemically and thermodynamically characterize them, specify their driving forces, and develop formalisms do describe all physical, information and chemical processes united in one phenomenon – life. However, partial chemical characterization (empirical formula) has been reported for just a few virus species [Jover et al., 2014]. A complete empirical formula was made for only one – the poliovirus [Molla et al., 1991]. Thermodynamic parameters of entire virus particles have never been determined. The parameter of particular interest is Gibbs energy, which represents the driving force of all processes in nature, including processes performed by organisms [Demirel, 2014; Von Stockar, 2014].

Some aspects of the viral life cycle have already been analyzed using thermodynamics. Katen and Zlotnick [2009] analyzed the thermodynamics of capsid assembly of several viruses, treating it as a polymerization reaction and providing new insights into the assembly mechanisms of spherical virus capsids, as well as into the biology of the viral life cycle. Ceres and Zlotnick [2002] used thermodynamics to analyze hepatitis B virus capsid assembly and found that it has a negative Gibbs energy change, implying that the process is thermodynamically spontaneous. Casasnovas and Springer [1995] studied the kinetics and thermodynamics of human rhinovirus interaction with its receptor, determining the enthalpy and Gibbs energy of their association, and analyzing their influence on virus disruption. Mahmoudabadi et al. [2017] developed a quantitative description of viral infection energetics, based on which they made predictions about viral evolution. Not only viral component synthesis and self-assembly were studied, Tzlil et al. [2004] made a statistical thermodynamic description of viral budding and found that complete budding (full wrapping of nucleocapsids) can only take place if the adhesion energy exceeds a certain, critical, bending Gibbs energy. However, there is still insufficient quantitative understanding of infection energetics [Mahmoudabadi et al., 2017].

Even though a lot has been discovered concerning the capability of viruses to rewire and undermine their host's metabolism [Shapshak et al., 2019], it is sometimes beneficial to develop simplified models that allow us to quantitatively describe viruses [Jones et al., 2015]. While these models lack a great amount of details, they are very useful due to their mathematical exactness. Jones et al. [2015] developed a model of the viral life cycle consisting of three phases: infection, immune clearance and reproduction. Based on this model, using the formalism of statistical mechanics and thermodynamics, they develop a mathematical description of viral life cycle and explain phenomena observed concerning viruses, such as the existence of various strategies used by viruses to avoid immune response and maximize their offspring number [Jones et al., 2015].

An important feature of the work described above is the use of the Gibbs [1902] formulation of statistical mechanics, through ensembles. Even though the Gibbs' statistical mechanics is a very powerful tool to analyze natural phenomena and is widely-used [McQuarrie, 2000; Sandler, 2010], it rests on certain assumptions, such as the ergodic hypothesis [Gibbs, 1902]. The ergodic hypothesis is briefly stated as *time average equals ensemble average* [Tolman, 1938]. The ergodic hypothesis applies to systems in equilibrium and can be applied to nonequilibrium steady state systems [Demirel, 2014]. However, multiplying viruses and cells increase their mass and volume during time and are thus nonequilibrium systems *out of steady state*. Thus, even though Gibbs’ statistical mechanics can give answers about many processes concerning organisms, like evolution, it cannot be applied to analyze their multiplication and growth. Thus, another approach had to be used in this work, that of nonequilibrium thermodynamics.

Nonequilibrium thermodynamics can be used to analyze growth of organisms and will be applied here to populations of viruses. The formalism of nonequilirium thermodynamics has been used to analyze bacterial growth, considering the process of bacterial multiplication as a single growth reaction [Von Stockar, 2014]. Similarly, since there are many viruses in an infected organism or microorganism colony, each of them will be in some phase of the viral life cycle. Thus, their contributions average out and the growth of a viral population can be summarized by a single growth reaction.

The influence of the medium on Gibbs energies has been shown in the literature for metabolic reactions [Meurer et al., 2016, 2017; Wangler et al., 2018; Greinert et al., 2020a, 2020b], but in this paper, this influence is neglected. Unlike bacteria, which can grow on various substrates, viruses are highly specific. Their growth is related to certain target cells that possess receptors that allow viruses penetration. In other words, viruses attack cells of certain tissues that in various organisms have the same composition. For example, cells of epithelium in various specimens of a species have the same chemical composition [Woodard and White, 1986].

The aim of this paper is to shed more light on viral multiplication and life cycle through nonequilibrium thermodynamic analysis. Thermodynamic characterization of viruses has first been made, followed by a comparison of Gibbs energies of growth of viruses and their host cells. The ratio of Gibbs energies of growth determines the growth rates of viruses and their hosts, and indicate the kind of cycle that viruses perform.

## 2 Methods

To describe the competition of viruses and their hosts using thermodynamics, several steps need to be made. First, elemental composition of viruses and their host cells needs to be known. These are necessary for the next two steps: to estimate their standard thermodynamic properties and to quantify their growth using growth reactions. Finally, these data are combined into thermodynamic properties of growth. More information on thermodynamic theory can be found in [Atkins and de Paula, 2011, 2014], while its application to organisms and growth reactions are described in [Von Stockar, 2014; Ozilgen and Sorgüven, 2017; Demirel, 2014].

### 2.1 Elemental composition of viruses, cells and tissues

Elemental compositions of human tissues, bacterial cells, and the poliovirus were taken from the literature [Bauer and Ziv, 1976; Duboc et al., 1999; Battley, 1992; Battley, 1998; Mayberry et al., 1968; Woodard and White, 1986; Molla et al., 1991; Dauner et al., 2001; Shastri and Morgan, 2008]. For the seven bacteriophages, hepatitis A and B viruses, polyoma virus, flock house virus, Saccharomyces cerevisiae viruses L-A and L-BC, elemental composition was calculated based on their structures taken from UniProt and NCBI (mode details in Supplementary Information 1 and 2), by counting the total number of atoms of each element in their genetic material and protein capsid. For the remaining viruses (herpes simplex, influenza, adenovirus), elemental composition was calculated from molecular composition. Elemental compositions of human tissues, bacterial cells, and viruses used for this study are given in Table 3.

Elemental compositions of living organisms are usually represented in two ways: mass fractions and empirical formulas. Mass fractions are often used in human body composition research, for example the composition of an average adult is 21.0% C, 10.2% H, 63.7% O, 2.7% N, 0.7% P, 0.2% S and 1.6% other elements [Wang et al., 1993]. Mass fractions are usually reported for hydrated live matter, live matter including water. Another means of expressing elemental composition of organisms are empirical formulas, also known as unit carbon formulas (UCF) or C-mole formulas [Battley, 2013]. UCFs express elemental composition of organisms as the number of each element present per mole of C. They are reported on a water-free basis, for an organism's dry mass. For example, the UCF of S. cerevisiae dry mass is CH_1.613_O_0.557_N_0.158_P_0.012_S_0.003_K_0.022_Mg_0.003_Ca_0.001_ [Battley, 1998]. The advantage of UCFs is that they represent the formula of an organism as a chemical compound and can easily be used to write growth reactions, which will be discussed below.

Elemental composition of human tissues was taken from Woodard and White [1986], who listed elemental composition of hydrated tissues in mass fraction form, as well as tissue water content (Table 4). To convert the data into UCFs, the composition of tissue dry matter had to be found first. For all elements except H and O, this was done by renormalizing their mass fractions to tissue mass without water

(1) $$w_{J}=w_{J,wet}/\left(1-w_{water}\right)$$

where *w*_*J*_ is the mass fraction of element *J* in cell dry matter, *w*_*J,wet*_ is the mass fraction of element *J* in hydrated cells, and *w*_*w*_ is the mass fraction of water in the cells. To find the mass fractions of H and O in tissue dry matter, a correction had to be made by subtracting H and O coming from water, using simple stoichiometry

(2) $$w_{H}=\left(w_{H,wet}-\frac{2}{18}w_{water}\right)/\left(1-w_{water}\right)$$

(3) $$w_{O}=\left(w_{O,wet}-\frac{16}{18}w_{water}\right)/\left(1-w_{water}\right)$$

The obtained element mass fractions in cell dry matter were then converted into mole fractions through the equation

(4) $$x_{J}=\left(\frac{w_{J}}{M_{r,J}}\right)/\left(\sum_{i}\frac{w_{i}}{M_{r,i}}\right)$$

where *x*_*J*_ is the mole fraction of element *J* in cell dry matter and *M*_*r,J*_ is the molar mass of element *J*. The summation is over all elements present in the cell. Finally, from the mole fraction data, unit-carbon formulas (UCF), were obtained by dividing the mole fraction of each element with that of carbon, *x*_*C*_,

(5) $$n_{J}=\frac{x_{J}}{x_{C}}$$

where *n*_*J*_ is the number of atoms of element *J* in the UCF.

For viruses of known capsid structure, elemental composition was calculated from capsid structure, capsid protein sequence and genetic code, as described in [Jover et al., 2014]. All analyzed viruses consisted of genetic material (DNA or RNA) packed in a protein capsid. The capsid typically consists of many copies of one or several kinds of proteins. Thus, if the number of copies of each protein is known, as well as its amino acid sequence, the number of atoms of each element in the capsid can be determined. Similarly, genetic material consists of one or several long chains made of four nucleotides (ATGC for DNA, in case of RNA U instead of T). Thus, using custom-made software, atoms of each element were counted and summed in all residues that comprise viral nucleic acid and protein sequences. In case of proteins, if there are several copies of a protein in the capsid, the number of atoms in a single protein is multiplied by the number of copies. The protein and nucleic acid sequences that comprise the analyzed viruses are given in Supplementary Information 1 and 2, respectively.

For viruses where the exact capsid structure was not known and viruses that contain lipids, molecular composition was used to determine empirical formulas, as described in [Wang et al., 1993]. Molecular constituents of viruses belong to five main categories: RNA, DNA, proteins, lipids and non-nucleic acid carbohydrates [Knight, 1975]. Virus molecular composition data was taken from the literature [Knight, 1975] in mass fraction form and converted into mole fractions using Eq. (4). The molar masses of the molecular constituents were obtained from their empirical formulas. The empirical formula of RNA was taken to be the average RNA of all RNA viruses considered in the atom counting method CH_1.2316_O_0.7610_N_0.3967_P_0.1050_, DNA was taken to be the average DNA of all DNA viruses considered in the atom counting method CH_1.2555_O_0.5840_N_0.3796_P_0.1022_, protein composition was taken as the average viral protein composition of all viruses considered in the atom counting method CH_1.5692_O_0.3085_N_0.2708_S_0.0061_, lipid composition was represented by that of human lipids CH_1.9216_O_0.1176_ [Wang et al., 1993] and non-nucleic acid carbohydrate composition was represented by the empirical formula of carbohydrates CH_2_O. The amount of each element in the virus empirical formula was found as

(6) $$n_{J}=\sum_{i}n_{J,i}x_{i}$$

where *n*_*J*_ is the number of atoms of element *J* in the virus empirical formula, *n*_*J,i*_ is the number of atoms of element *J* in the empirical formula of the molecular constituent *i* and *x*_*i*_ is the mole fraction of constituent *i* in the virus. The summation is over five classes of molecular constituents.

To ensure the data is consistent, elemental compositions of the poliovirus were obtained using both described methods and compared to the literature experimental value [Molla et al., 1991]. The content of elements that influence thermodynamic properties the most C, H, O and N, was in agreement to 2% accuracy.

### 2.2 Thermodynamic properties of life matter

Based on elemental composition thermodynamic properties of live matter can be calculated in two ways: using the Battley and Roels methods. Both methods are reviewed below. Since the Battley method is more precise [Von Stockar and Liu, 1999], it was used to calculate thermodynamic properties of live matter presented in the Results and Discussion section. The Roels method was used to check whether changing the model for estimating thermodynamic properties will influence the conclusions of the paper.

#### 2.2.1 Battley method

Thermodynamic properties of viruses and cells were calculated from elemental composition, as described in [Popovic, 2019]. Elemental composition of animate matter can be used to determine its enthalpy of formation through the Patel-Erickson equation and classical reaction thermochemistry. The Patel-Erickson equation is based on the fact that heat released during combustion is proportional to number of electrons transferred to oxygen. For live matter, the Patel-Erickson equation takes the form [Patel and Erickson, 1981; Battley, 1998]

(7) $$\Delta_{C}H^{0}=-111.14\frac{kJ}{mol}\cdot E$$

where Δ_*C*_*H⁰* is standard enthalpy of combustion of live matter and *E* is the number of electrons transferred to oxygen during combustion to CO_2_(g), H_2_O(l), N_2_(g), P_4_O_10_(s) and SO_3_(g) (for a discussion on other conventions concerning SO_3_ please see [Popovic, 2019]). During combustion, a C atom gives its 4 valence electrons to O, H gives 1, N gives none since it is converted to N_2_, P gives 5 and S gives 6. Inorganic ions, like Na^+^ and Mg^2+^ are not included, since they are already in their highest oxidation state and cannot transfer any electrons to oxygen [Battley, 1998]. Thus, *E* is calculated through the equation

(8) $$E=4\;n_{C}+n_{H}-2\;n_{O}-0\;n_{N}+5\;n_{P}+6\;n_{S}$$

where *n*_*C*_, *n*_*H*_, *n*_*O*_, *n*_*N*_, *n*_*P*_ and *n*_*S*_ are the number of C, H, O, N, P and S atoms in the biomass empirical formula [Patel and Erickson, 1981; Battley, 1998]. If any of these atoms are not present, they are just neglected during the calculation [Battley, 1998].

The process of combustion of live matter can be represented by a general chemical reaction of the form

(9) CnCHnHOnONnNPnPSnSNanNaKnKMgnMgCanCaClnCl+(nC+14nH+114nP+112nS+ 14nNa+14nK+12nMg+12nCa+34nFe−12nO−14 nCl)O2→nCCO2+12nHH2O+12nNN2+14nPP4O10+nSSO3+12nNaNa2O+12nKK2O+nMgMgO+nCaCaO+nClHCl

where the formula C_nC_H_nH_O_nO_N_nN_P_nP_S_nS_Na_nNa_K_nK_Mg_nMg_Ca_nCa_Cl_nCl_ represents live matter. Standard enthalpy of combustion of live matter Δ_*C*_*H⁰*, that is the enthalpy of reaction (9), can be found through simple thermochemistry [Atkins and de Paula, 2014], as the difference between standard enthalpies of formation, Δ_*f*_*H⁰*, of products and reactants

(10) $$\Delta_{C}H^{0}=n_{C}\Delta_{f}H^{0}\left(CO_{2}\right)+\frac{1}{2}\left(n_{H}-n_{Cl}\right)\Delta_{f}H^{0}\left(H_{2}O\right)+\frac{1}{4}n_{P}\Delta_{f}H^{0}\left(P_{4}O_{10}\right)+n_{S}\Delta_{f}H^{0}\left(SO_{3}\right)+\frac{1}{2}n_{Na}\Delta_{f}H^{0}\left(Na_{2}O\right)+\frac{1}{2}n_{K}\Delta_{f}H^{0}\left(K_{2}O\right)+n_{Mg}\Delta_{f}H^{0}\left(MgO\right)+n_{Ca}\Delta_{f}H^{0}\left(CaO\right)+n_{Cl}\Delta_{f}H^{0}\left(HCl\right)-\Delta_{f}H^{0}\left(bio\right)$$

where Δ_*f*_*H⁰*(*bio*) is standard enthalpy of formation of live matter. While deriving Eq. (10), enthalpies of formation of elemental O_2_ and N_2_ were neglected, since they are by definition zero [Atkins and de Paula, 2014]. Eq. (10) can be rearranged to yield Δ_*f*_*H⁰*(*bio*)

(11) $$\Delta_{f}H^{0}\left(bio\right)=n_{C}\Delta_{f}H^{0}\left(CO_{2}\right)+\frac{1}{2}\left(n_{H}-n_{Cl}\right)\Delta_{f}H^{0}\left(H_{2}O\right)+\frac{1}{4}n_{P}\Delta_{f}H^{0}\left(P_{4}O_{10}\right)+n_{S}\Delta_{f}H^{0}\left(SO_{3}\right)+\frac{1}{2}n_{Na}\Delta_{f}H^{0}\left(Na_{2}O\right)+\frac{1}{2}n_{K}\Delta_{f}H^{0}\left(K_{2}O\right)+n_{Mg}\Delta_{f}H^{0}\left(MgO\right)+n_{Ca}\Delta_{f}H^{0}\left(CaO\right)+n_{Cl}\Delta_{f}H^{0}\left(HCl\right)-\Delta_{C}H^{0}$$

Elemental composition can also be used to determine standard molar entropy of live matter, *S⁰*_*m*_ (*bio*), through the Battley equation [Battley, 1999]

(12) $$S_{m}^{o}\left(bio\right)=0.187\sum_{J}\frac{S_{m}^{o}\left(J\right)}{a_{J}}n_{J}$$

where *n*_*J*_ is the number of atoms of element *J* in the empirical formula of the biomass, *S⁰*_*m*_(*J*) is standard molar entropy of element *J* and *a*_*J*_ is the number of atoms per molecule of element *J* in its standard state elemental form. For example, the standard state elemental form of carbon is graphite, which is simply written as C, which makes *a*_*C*_ = 1. On the other hand, hydrogen, oxygen and nitrogen are in their standard state elemental forms all diatomic gasses H_2_, O_2_ and N_2_, respectively, which implies that *a*_*H*_ = *a*_*O*_ = *a*_*N*_ = 2. The summation is over all elements constituting the dry live matter. The Battley equation can also be used to determine standard entropy *of formation* of live matter Δ_*f*_*S⁰*(*bio*). In this case, it takes the form [Battley, 1999]

(13) $$\Delta_{f}S^{0}\left(bio\right)=-0.813\sum_{J}\frac{S_{m}^{o}\left(J\right)}{a_{J}}n_{J}$$

Finally, by combining standard enthalpy and entropy of formation, it is possible to calculate standard Gibbs energy of formation of live matter, Δ_*f*_*G⁰*(*bio*), as

(14) $$\Delta_{f}G^{0}\left(bio\right)=\Delta_{f}H^{0}\left(bio\right)-T\;\Delta_{f}S^{0}\left(bio\right)$$

where *T* is temperature. Standard thermodynamic properties of living organisms are reported in Table 1.

**Table 1:** Standard thermodynamic properties of formation of viruses and their host cells and tissues. Δ_*f*_*H⁰* and Δ_*f*_*G⁰* are standard enthalpy and Gibbs energy of formation, respectively. *S⁰*_*m*_ is standard molar entropy.

Name	Δ_f_H⁰_bio_ (kJ/C-mol)	S⁰_m,bio_ (J/C-mol K)	Δ_f_G⁰_bio_ (kJ/C-mol)
Poliovirus	-86.17 ± 28.87	32.19 ± 6.34	-44.45 ± 30.76
Gastrointestinal tract - small intestine (wall)	-53.40 ± 31.17	29.00 ± 5.71	-15.82 ± 32.87
Brain-grey matter	-71.79 ± 32.76	32.83 ± 6.47	-29.23 ± 34.69

Hepatovirus A	-90.60 ± 28.55	32.23 ± 6.35	-48.82 ± 30.44
Hepatovirus B	-75.82 ± 29.57	32.03 ± 6.31	-34.30 ± 31.45
Liver	-69.58 ± 30.44	30.88 ± 6.08	-29.55 ± 32.25

Human herpes virus (entire virus)	-63.88 ± 30.72	30.21 ± 5.95	-24.72 ± 32.49
Human herpes virus 1 (no envelope)	-121.41 ± 27.94	34.55 ± 6.81	-76.62 ± 29.97
Epithelium∗	-65.61 ± 30.74	31.37 ± 6.18	-24.94 ± 32.59
Neurons∗	-71.79 ± 32.76	32.83 ± 6.47	-29.23 ± 34.69

Influenza	-66.05 ± 30.50	30.55 ± 6.02	-26.45 ± 32.29
Adenovirus	-72.85 ± 29.49	31.41 ± 6.19	-32.13 ± 31.34
Lung - parenchyma	-65.61 ± 30.74	31.37 ± 6.18	-24.94 ± 32.59

BK polyomavirus (BKPyV) (Human polyomavirus 1)	-73.13 ± 29.64	31.66 ± 6.24	-32.08 ± 31.50
Kidney	-61.19 ± 31.06	29.74 ± 5.86	-22.64 ± 32.81

Flock house virus	-75.98 ± 29.37	31.92 ± 6.29	-34.61 ± 31.25
Saccharomyces cerevisiae virus L-A	-74.09 ± 29.30	30.95 ± 6.10	-33.97 ± 31.12
Saccharomyces cerevisiae virus L-BC	-75.87 ± 29.41	31.38 ± 6.18	-35.20 ± 31.25
Saccharomyces cerevisiae	-131.99 ± 27.27	34.66 ± 6.83	-87.07 ± 29.30

Enterobacteria phage T4	-94.44 ± 28.77	32.59 ± 6.42	-52.20 ± 30.68
Enterobacteria phage N4	-101.41 ± 28.63	33.14 ± 6.53	-58.46 ± 30.58
Enterobacteria phage T7	-103.79 ± 28.68	33.60 ± 6.62	-60.23 ± 30.66
Enterobacteria phage lambda	-102.54 ± 28.58	33.19 ± 6.54	-59.51 ± 30.53
Enterobacteria phage PRD1 (Bacteriophage PRD1)	-78.00 ± 29.13	31.40 ± 6.19	-37.29 ± 30.97
Escherichia coli	-98.94 ± 28.82	34.32 ± 6.76	-54.46 ± 30.84

Enterobacteria phage PRD1 (Bacteriophage PRD1)	-78.00 ± 29.13	31.40 ± 6.19	-37.29 ± 30.97
Pseudomonas C12B	-128.09 ± 29.55	39.56 ± 7.79	-76.80 ± 31.87

Bacillus phage phi29	-93.83 ± 28.82	32.73 ± 6.45	-51.41 ± 30.75
Bacillus subtilis	-87.49 ± 29.39	32.48 ± 6.40	-45.40 ± 31.30

Cyanophage Syn5 virus	-106.26 ± 28.39	33.49 ± 6.60	-62.86 ± 30.36
Cyanobacteria Synechocystis PCC 6803	-88.41 ± 28.44	31.39 ± 6.18	-47.73 ± 30.28

∗ The properties of epithelium and neurons were set equal to Lung – parenchyma and Brain-grey matter, respectively, since these tissues are made primarily of the corresponding cells.

#### 2.2.2 Roels method

Except for the method described above, Gibbs energy of live matter can be determined using the Roels equation. The Roels equation is analogous to the Patel-Erickson equation, giving standard Gibbs energy of combustion, Δ_*C*_*G⁰*, of live matter

(27) $$\Delta_{C}G^{0}=-86.6\frac{kJ}{mol}-94.4\frac{kJ}{mol}\cdot E$$

where *E* is the number of electrons transferred to oxygen during combustion to CO_2_(g), H_2_O(l), N_2_(g), P_4_O_10_(s) and SO_3_(g) [Roels, 1983; Von Stockar and Liu, 1999]. Gibbs energy of combustion of live matter is the Gibbs energy change of reaction (9). Thus, since live matter is a reactant in reaction (9), its Gibbs energy can be determined from Δ_*C*_*G⁰* using an equation analogous to Eq. (11)

(28) $$\Delta_{f}G^{0}\left(bio\right)=n_{C}\Delta_{f}G^{0}\left(CO_{2}\right)+\frac{1}{2}\left(n_{H}-n_{Cl}\right)\Delta_{f}G^{0}\left(H_{2}O\right)+\frac{1}{4}n_{P}\Delta_{f}G^{0}\left(P_{4}O_{10}\right)+n_{S}\Delta_{f}G^{0}\left(SO_{3}\right)+\frac{1}{2}n_{Na}\Delta_{f}G^{0}\left(Na_{2}O\right)+\frac{1}{2}n_{K}\Delta_{f}G^{0}\left(K_{2}O\right)+n_{Mg}\Delta_{f}G^{0}\left(MgO\right)+n_{Ca}\Delta_{f}G^{0}\left(CaO\right)+n_{Cl}\Delta_{f}G^{0}\left(HCl\right)-\Delta_{C}G^{0}$$

**Figure 1:**
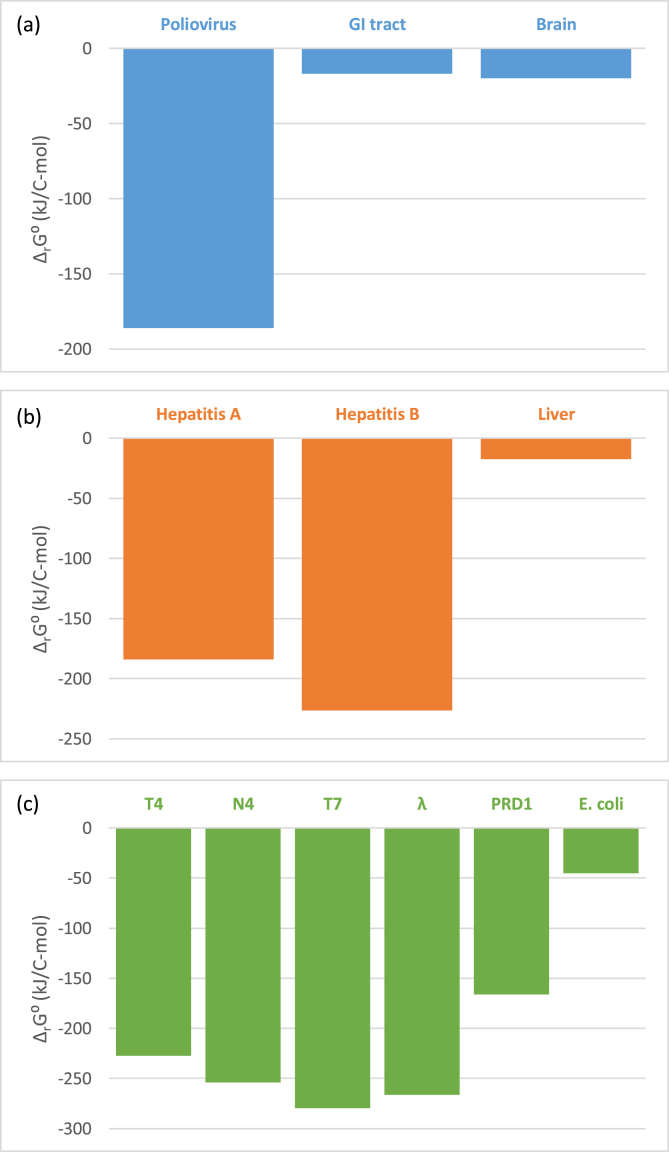
Comparison of Gibbs energies of growth of viruses and their host tissues. GI tract stands for Gastrointestinal tract - small intestine (wall), Brain for Brain-grey matter, while T4, N4, T7, λ and PRD1 stand for Enterobacteria phages T4, N4, T7, λ and PRD1, respectively.

The Roels method and the Battley method are complementary ways of finding Gibbs energy of formation of live matter. However, the Battley equation was calibrated on a better dataset than the Roels equation, making it more precise [Von Stockar and Liu, 1999]. Thus, all results presented in Tables 1 and 2, and Figure 1 are based on the Battley method. The Roels method was used to make a parallel calculation of Δ_*f*_*G⁰*(*bio*), to determine whether the conclusions of this research are dependent on the method used to find live matter thermodynamic properties.

**Table 2:** Standard thermodynamic properties of growth of viruses and their host cells and tissues. Δ_*r*_*H⁰*, Δ_*r*_*S⁰* and Δ_*r*_*G⁰* are standard reaction enthalpy, entropy and Gibbs energy, respectively.

Name	Δ_r_H⁰ (kJ/mol)	Δ_r_S⁰ (J/mol K)	Δ_r_G⁰ (J/mol K)
Poliovirus	-195.75 ± 28.87	-33.05 ± 6.34	-186.14 ± 30.76
Gastrointestinal tract - small intestine (wall)	-11.68 ± 31.17	18.34 ± 5.71	-16.85 ± 32.87
Brain-grey matter	-13.05 ± 32.76	23.99 ± 6.47	-19.86 ± 34.69

Hepatovirus A	-193.79 ± 28.55	-33.57 ± 6.35	-184.07 ± 30.44
Hepatovirus B	-237.42 ± 29.57	-38.06 ± 6.31	-226.28 ± 31.45
Liver	-15.51 ± 30.44	6.38 ± 6.08	-17.42 ± 32.25

Human herpes virus (entire virus)	-9.73 ± 30.72	13.60 ± 5.95	-13.64 ± 32.49
Human herpes virus 1 (no envelope)	-392.78 ± 27.94	-72.66 ± 6.81	-371.99 ± 29.97
Epithelium∗	-50.51 ± 30.74	-2.80 ± 6.18	-49.76 ± 32.59
Neurons∗	-13.05 ± 32.76	23.99 ± 6.47	-19.86 ± 34.69

Influenza	-10.29 ± 30.50	13.02 ± 6.02	-13.95 ± 32.29
Adenovirus	-150.32 ± 29.49	-22.42 ± 6.19	-143.73 ± 31.34
Lung - parenchyma	-50.51 ± 30.74	-2.80 ± 6.18	-49.76 ± 32.59

BK polyomavirus (BKPyV) (Human polyomavirus 1)	-167.93 ± 29.64	-25.57 ± 6.24	-160.43 ± 31.50
Kidney	-9.01 ± 31.06	16.44 ± 5.86	-13.72 ± 32.81

Flock house virus	-189.35 ± 29.37	-30.01 ± 6.29	-180.52 ± 31.25
Saccharomyces cerevisiae virus L-A	-160.76 ± 29.30	-24.80 ± 6.10	-153.56 ± 31.12
Saccharomyces cerevisiae virus L-BC	-158.73 ± 29.41	-24.56 ± 6.18	-151.61 ± 31.25
Saccharomyces cerevisiae	-14.81 ± 27.27	4.12 ± 6.83	-15.90 ± 29.30

Enterobacteria phage T4	-239.19 ± 28.77	-41.36 ± 6.42	-227.32 ± 30.68
Enterobacteria phage N4	-267.65 ± 28.63	-47.37 ± 6.53	-254.10 ± 30.58
Enterobacteria phage T7	-294.88 ± 28.68	-52.35 ± 6.62	-279.87 ± 30.66
Enterobacteria phage lambda	-280.34 ± 28.58	-49.77 ± 6.54	-266.09 ± 30.53
Enterobacteria phage PRD1 (Bacteriophage PRD1)	-173.93 ± 29.13	-27.61 ± 6.19	-165.89 ± 30.97
Escherichia coli	-47.43 ± 28.82	-7.27 ± 6.76	-45.25 ± 30.84

Enterobacteria phage PRD1 (Bacteriophage PRD1)	-173.93 ± 29.13	-27.61 ± 6.19	-165.89 ± 30.97
Pseudomonas C12B	-19.74 ± 29.55	-3.58 ± 7.79	-18.67 ± 31.87

Bacillus phage phi29	-233.82 ± 28.82	-40.21 ± 6.45	-222.25 ± 30.75
Bacillus subtilis	-32.42 ± 29.39	-2.79 ± 6.40	-31.75 ± 31.30

Cyanophage Syn5 virus	-300.29 ± 28.39	-53.96 ± 6.60	-284.82 ± 30.36
Cyanobacteria Synechocystis PCC 6803	-12.47 ± 28.44	4.78 ± 6.18	-13.74 ± 30.28

∗ The properties of epithelium and neurons were set equal to Lung – parenchyma and Brain-grey matter, respectively, since these tissues are made primarily of the corresponding cells.

**Table 3:** Elemental compositions of analyzed viruses, cells and tissues. The data is given in the empirical formula format, normalized per C mole. For example, the empirical formula of the poliovirus is CH_1.4802_O_0.3944_N_0.2953_P_0.0225_S_0.0070_. In the Source column, “atom counting” and “molecular composition” stand for the atom counting and molecular composition methods, respectively, described in the Methods section.

Name	Live matter composition											Source
	C	H	O	N	P	S	Na	K	Mg	Ca	Cl	
Poliovirus	1	1.4802	0.3944	0.2953	0.0225	0.0070	0	0	0	0	0	Molla et al. (1991)
Gastrointestinal tract - small intestine (wall)	1	1.6480	0.2310	0.1789	0.0028	0.0054	0.0038	0.0044	0	0	0.0024	Woodard and White (1986)
Brain-grey matter	1	1.9096	0.2590	0.1625	0.0122	0.0079	0.0110	0.0097	0	0	0.0107	Woodard and White (1986)

Hepatovirus A	1	1.4588	0.4128	0.2922	0.0254	0.0053	0	0	0	0	0	Atom counting
Hepatovirus B	1	1.5210	0.3424	0.3163	0.0195	0.0051	0	0	0	0	0	Atom counting
Liver 1	1	1.6480	0.2904	0.1851	0.0084	0.0081	0.0075	0.0066	0	0	0.0049	Woodard and White (1986)
Liver 2	1	1.6079	0.3277	0.2246	0.0092	0.0089	0.0083	0.0073	0	0	0.0054	Woodard and White (1986)
Liver 3	1	1.5863	0.2866	0.2462	0.0077	0.0111	0.0103	0.0061	0	0	0.0101	Woodard and White (1986)

Human herpes virus (entire virus)	1	1.6598	0.2789	0.1939	0.0063	0.0038	0	0	0	0	0	Molecular composition
Human herpes virus 1 (no envelope)	1	1.3394	0.5202	0.3674	0.0757	0.0022	0	0	0	0	0	Atom counting
Epithelium∗	1	1.6268	0.2836	0.2532	0.0074	0.0107	0.0100	0.0059	0	0	0.0097	Woodard and White (1986)
Neurons∗	1	1.9096	0.2590	0.1625	0.0122	0.0079	0.0110	0.0097	0	0	0.0107	Woodard and White (1986)

Influenza	1	1.6751	0.2920	0.1911	0.0006	0.0043	0	0	0	0	0	Molecular composition
Adenovirus	1	1.5386	0.3354	0.2814	0.0100	0.0055	0	0	0	0	0	Molecular composition
Lung - parenchyma	1	1.6268	0.2836	0.2532	0.0074	0.0107	0.0100	0.0059	0	0	0.0097	Woodard and White (1986)

BK polyomavirus (BKPyV) (Human polyomavirus 1)	1	1.5396	0.3382	0.2897	0.0122	0.0085	0	0	0	0	0	Atom counting
Kidney 1	1	1.6151	0.2452	0.1949	0.0059	0.0057	0.0079	0.0047	0	0.0023	0.0051	Woodard and White (1986)
Kidney 2	1	1.6364	0.2581	0.2184	0.0073	0.0071	0.0099	0.0058	0	0.0028	0.0064	Woodard and White (1986)
Kidney 3	1	1.6891	0.2593	0.1484	0.0075	0.0077	0.0067	0.0059	0	0	0.0043	Woodard and White (1986)

Flock house virus	1	1.5343	0.3498	0.2959	0.0115	0.0064	0	0	0	0	0	Atom counting
Saccharomyces cerevisiae virus L-A	1	1.4781	0.3435	0.2845	0.0180	0.0063	0	0	0	0	0	Atom counting
Saccharomyces cerevisiae virus L-BC	1	1.5000	0.3509	0.2847	0.0178	0.0082	0	0	0	0	0	Atom counting
Saccharomyces cerevisiae	1	1.6130	0.5570	0.1580	0.0120	0.0030	0	0.0220	0.0030	0.0010	0	Battley (1998)

Enterobacteria phage T4	1	1.4445	0.4167	0.3120	0.0398	0.0032	0	0	0	0	0	Atom counting
Enterobacteria phage N4	1	1.4256	0.4436	0.3226	0.0489	0.0039	0	0	0	0	0	Atom counting
Enterobacteria phage T7	1	1.4347	0.4505	0.3337	0.0517	0.0038	0	0	0	0	0	Atom counting
Enterobacteria phage lambda	1	1.4174	0.4470	0.3271	0.0511	0.0031	0	0	0	0	0	Atom counting
Enterobacteria phage PRD1 (Bacteriophage PRD1)	1	1.4920	0.3558	0.2881	0.0177	0.0034	0	0	0	0	0	Atom counting
Escherichia coli	1	1.7700	0.4900	0.2400	0	0	0	0	0	0	0	Bauer and Ziv (1976)
E. coli	1	1.7400	0.3400	0.2200	0	0	0	0	0	0	0	Duboc et al. (1999)
E. coli K-12: grown on Acetic acid	1	1.5400	0.4000	0.2100	0	0	0	0	0	0	0	Battley (1992)
E. coli K-12: grown on glucose	1	1.7400	0.4640	0.2600	0	0	0	0	0	0	0	Battley (1992)
E. coli K-12: grown on glucose	1	1.8100	0.4000	0.2200	0	0	0	0	0	0	0	Battley (1992)
E. coli K-12: grown on glucose	1	1.7300	0.5300	0.2350	0	0	0	0	0	0	0	Battley (1992)
E. coli K-12: grown on glucose	1	1.7800	0.5110	0.2370	0	0	0	0	0	0	0	Battley (1992)
E. coli K-12: grown on glucose	1	1.8100	0.4900	0.2340	0	0	0	0	0	0	0	Battley (1992)
E. coli K-12: grown on glucose	1	1.5400	0.3400	0.2400	0	0	0	0	0	0	0	Battley (1992)
E. coli K-12: grown on Succinic acid	1	1.5600	0.3600	0.2300	0	0	0	0	0	0	0	Battley (1992)
E. coli W: grown on glucose	1	1.6980	0.4270	0.2500	0	0	0	0	0	0	0	Battley (1992)
E. coli W: grown on glycerol	1	1.6980	0.4270	0.2500	0	0	0	0	0	0	0	Battley (1992)

Enterobacteria phage PRD1 (Bacteriophage PRD1)	1	1.4920	0.3558	0.2881	0.0177	0.0034	0	0	0	0	0	Atom counting
Pseudomonas C12B	1	2.0000	0.5200	0.2300	0	0	0	0	0	0	0	Mayberry et al. (1968)

Bacillus phage phi29	1	1.4666	0.4131	0.3101	0.0365	0.0026	0	0	0	0	0	Atom counting
B. subtilis (P-limited growth, 0.1h^−1^)	1	1.6080	0.3640	0.2350	0.0080	0.0060	0	0	0	0	0	Dauner et al. (2001)
B. subtilis (P-limited growth, 0.4h^−1^)	1	1.5940	0.3870	0.2390	0.0120	0.0050	0	0	0	0	0	Dauner et al. (2001)
B. subtilis (N-limited growth, 0.4h^−1^)	1	1.6260	0.4120	0.2310	0.0210	0.0050	0	0	0	0	0	Dauner et al. (2001)

Cyanophage Syn5 virus	1	1.4074	0.4618	0.3336	0.0538	0.0023	0	0	0	0	0	Atom counting
Cyanobacteria Synechocystis PCC 6803	1	1.5772	0.4019	0.1884	0	0	0	0	0	0	0	Shastri and Morgan (2008)

∗ The compositions of epithelium and neurons were set equal to Lung – parenchyma and Brain-grey matter, respectively, since these tissues are made primarily of the corresponding cells.

**Table 4:** Elemental composition and water content of hydrated human tissues. All values are in mass fractions. Data taken from Woodard and White [1986].

Tissue	C	H	O	N	P	S	Na	K	Ca	Cl	Water
Gastrointestinal tract - small intestine (wall)	11.5	10.6	75.1	2.2	0.1	0.1	0.1	0.1	0.0	0.2	80.6
Brain-grey matter	9.5	10.7	76.7	1.8	0.3	0.2	0.2	0.3	0.0	0.3	82.6
Liver 1	15.6	10.3	70.1	2.7	0.3	0.3	0.2	0.3	0.0	0.2	72.8
Liver 2	13.9	10.2	71.6	3.0	0.3	0.3	0.2	0.3	0.0	0.2	74.5
Liver 3	12.6	10.1	72.7	3.3	0.3	0.3	0.2	0.3	0.0	0.2	75.6
Lung - parenchyma	10.1	10.3	75.5	2.9	0.2	0.3	0.2	0.2	0.0	0.3	80.6
Kidney 1	16.0	10.2	69.3	3.4	0.2	0.2	0.2	0.2	0.1	0.2	72.3
Kidney 2	13.2	10.3	72.4	3.0	0.2	0.2	0.2	0.2	0.1	0.2	76.6
Kidney 3	10.6	10.4	75.2	2.7	0.2	0.2	0.2	0.2	0.1	0.2	80.5

### 2.3 Growth reactions

Based on empirical formulas of tissues, cells and viruses, chemical reactions can be formulated that represent growth of living organisms, called growth reactions [Battley, 1998; Von Stockar, 2014]. To grow, an organism needs sources of several key elements: C, N, P and S. Each of these elements can come from a different compound, C from glucose, N from an ammonium salt, P from phosphates and S from sulfates. Also, several elements can come from a single compound, for example methionine (C_5_H_11_O_2_NS) is simultaneously a source of C, N and S. For this research, the composition resembling that of human blood plasma was chosen as the growth medium, since it can support growth of all the analyzed host cells and tissues. This allows the results for various hosts to be compared without any influence of growth medium difference. An equimolar mixture of amino acids was chosen as the source of N, S and partly C, with the empirical formula CH_1.7978_O_0.4831_N_0.2247_S_0.0225_. Since C is necessary in greater amount than provided by the amino acid, the remaining carbon comes from glucose, with the empirical formula CH_2_O. The source of P was the hydrogen phosphate ion HPO_4_^2-^. The sources of inorganic ions were Na^+^, K^+^, Mg^2+^, Ca^2+^ and Cl^−^. Since S in amino acids come in a quantity greater than needed for growth, the excess S is removed as the SO_4_^2-^ ion. The pH of the growth mixture is regulated by the bicarbonate buffer. Thus, the general unbalanced growth reaction has the form

(15) $${\text{CH}}_{1.7978}{\text{O}}_{0.4831}{\text{N}}_{0.2247}{\text{S}}_{0.0225}+{\text{CH}}_{2}\text{O}+{\text{O}}_{2}+\text{HP}{{\text{O}}_{4}}^{2-}+{\text{H}}_{2}\text{O}+{\text{HCO}}_{3^{-}}+{\text{Na}}^{+}+{\text{K}}^{+}+{\text{Mg}}^{2+}+{\text{Ca}}^{2+}+{\text{Cl}}^{-}\to \left(\text{Bio}\right)\;+\text{ S}{{\text{O}}_{4}}^{2-}+{\text{H}}_{2}\text{O}+{\text{HCO}}_{3^{-}}+{\text{H}}_{2}{\text{CO}}_{3}$$

where *Bio* denotes live matter. The species H_2_O and HCO_3_^-^ appear on both sides, because they maintain the O and H balances, respectively. Since O and H balances can vary greatly from reaction to reaction H_2_O and HCO_3_^-^ can be either reactants or products of a reaction. The composition of live matter varies from species to species and is given in Table 1. The growth reaction stoichiometric coefficients of all analyzed species are given in Supplementary Information 3.

Growth reaction thermodynamic parameters were calculated using classical thermochemistry

(16) $$\Delta_{r}H^{0}=\sum_{products}\nu \;\Delta_{f}H^{0}-\sum_{reactants}\nu \;\Delta_{f}H^{0}$$

(17) $$\Delta_{r}S^{0}=\sum_{products}\nu \;S_{m}^{o}-\sum_{reactants}\nu \;\;S_{m}^{o}$$

(18) $$\Delta_{r}G^{0}=\sum_{products}\nu \;\Delta_{f}G^{0}-\sum_{reactants}\nu \;\Delta_{f}G^{0}$$

where ν′s are stoichiometric coefficients of species participating the reaction, while Δ_*r*_*H⁰*, Δ_*r*_*S⁰* and Δ_*r*_*G⁰* are standard enthalpy, entropy and Gibbs energy of growth, respectively [Atkins and de Paula, 2014, 2011]. The calculated values of Δ_*r*_*H⁰*, Δ_*r*_*S⁰* and Δ_*r*_*G⁰* for all the analyzed species are given in Table 2.

### 2.4 Uncertainties

Thermodynamic properties were determined from elemental composition using empirical relations and thus have some uncertainty. Δ_*C*_*H⁰* was found using the Patel-Erickson equation, the uncertainty of which is 5.36% [Popovic, 2019]. The determined Δ_*C*_*H⁰* values were then subtracted from standard enthalpies of formation of oxides (Eq. (11)) to find Δ_*f*_*H⁰*(*bio*). Since standard enthalpies of formation of oxides were precisely determined by experiment (more details in [Chase, 1998]), they have a negligible error compared to that in Δ_*C*_*H⁰*. Thus, the uncertainty in standard enthalpy of formation of live matter, *δ*(Δ_*f*_*H⁰*(*bio*)), is equal to the error in Δ_*C*_*H⁰*.

(19) $$\delta \left(\Delta_{f}H^{0}\left(bio\right)\right)=0.0536\cdot \left|-111.14\frac{kJ}{mol}\left(4\;n_{C}+n_{H}-2\;n_{O}-0\;n_{N}+5\;n_{P}+6\;n_{S}\right)\right|$$

*S⁰*_*m*_ (*bio*) was determined using the Battley equation, which was calibrated on a wide range of organic molecule and live matter data [Battley, 1999]. The uncertainty in estimation of entropy using the Battley equation is 2% for dry matter and 19.7% for hydrated matter [Battley, 1999]. Therefore, the uncertainty in standard molar entropy of live matter, *δ*(*S⁰*_*m*_ (*bio*)), is

(20) $$\delta (S_{m}^{0}\left(bio\right))=0.197\cdot S_{m}^{0}\left(bio\right)$$

Δ_*f*_*S⁰*(*bio*) is the entropy of the reaction

(21) $${\text{n}}_{\text{c}}\text{C}+\text{½ }{\text{n}}_{\text{H}}{\text{H}}_{2}+\text{½ }{\text{n}}_{\text{O}}{\text{O}}_{2}+\text{½ }{\text{n}}_{\text{N}}{\text{N}}_{2}+{\text{n}}_{\text{P}}\text{P}+{\text{n}}_{\text{S}}\text{S}+{\text{n}}_{\text{Na}}\text{Na}+{\text{n}}_{\text{K}}\text{K}+{\text{n}}_{\text{Mg}}\text{Mg}+{\text{n}}_{\text{Ca}}\text{Ca}+{\text{½n}}_{\text{Cl}}\text{Cl}\to {\text{C}}_{\text{nC}}{\text{H}}_{\text{nH}}{\text{O}}_{\text{nO}}{\text{N}}_{\text{nN}}{\text{P}}_{\text{nP}}{\text{S}}_{\text{nS}}{\text{Na}}_{\text{nNa}}{\text{K}}_{\text{nK}}{\text{Mg}}_{\text{nMg}}{\text{Ca}}_{\text{nCa}}{\text{Cl}}_{\text{nCl}}$$

and is defined as the difference in *S⁰*_*m*_ (*bio*) and standard molar entropies of the elements, which have been determined with great accuracy by experiment [Chase, 1998]. Thus, the uncertainty in Δ_*f*_*S⁰*(*bio*) is equal to that in *S⁰*_*m*_ (*bio*) [Popovic, 2019].

Δ_*f*_*H⁰*(*bio*) and Δ_*f*_*S⁰*(*bio*) are used to find Δ_*f*_*G⁰*(*bio*). Therefore, the uncertainty in the standard Gibbs energy of formation of live matter, *δ*(Δ_*f*_*G⁰*(*bio*)), is [Popovic, 2019]

(22) $$\delta \left(\Delta_{f}G^{0}\left(bio\right)\right)=\delta \left(\Delta_{f}H^{0}\left(bio\right)\right)+T\cdot \delta (S_{m}^{0}\left(bio\right))$$

Finally, the uncertainty in Δ_*f*_*G⁰*(*bio*) is equal to that in Δ_*r*_*G⁰*, since it is the greatest source of uncertainty in its determination. Δ_*r*_*G⁰* is determined using Eq. (18), as the difference of Δ_*f*_*G⁰* values of reactants and products. The Δ_*f*_*G⁰* values of all reaction participants, except for live matter have been determined with great accuracy by experiment [Chase, 1998]. Thus, uncertainty in Gibbs energy of growth, *δ*(Δ_*r*_*G⁰*), is equal to *δ*(Δ_*f*_*G⁰*(*bio*)). Similarly, *δ*(Δ_*r*_*H⁰*) and *δ*(Δ_*r*_*S⁰*) are equal to *δ*(Δ_*f*_*H⁰*(*bio*)) and *S⁰*_*m*_ (*bio*), respectively.

## 3 Results and Discussion

In this research, standard thermodynamic properties of 17 viruses, and their host cells and tissues, were calculated (Table 1), including 7 human viruses, 3 yeast viruses and 7 bacteriophages. For all analyzed viruses and hosts, standard enthalpies and Gibbs energies of formation are negative, while all standard molar entropies are positive.

A virus hijacks metabolic pathways of its host cell, making viral multiplication predominant and performing the lytic cycle [Walsh and Mohr, 2011]. The biosynthesis of viral and host cell components is competitive, since they use the same precursors. To understand the competition, it is necessary to quantitatively compare their standard Gibbs energies of growth. If the Gibbs energy of growth is lower for a virus than its host, then it can make the growth reaction rate of a virus greater (Eq. (23)), implying more efficient multiplication and virus growth, as will be shown below.

As discussed in Section 2.3, chemical reactions can be used to represent growth of living organisms, called growth reactions [Battley, 1998; Von Stockar, 2014]. The rate of a growth reaction, *r*, can be related to Gibbs energy of growth, Δ_*r*_*G*, using nonequilibrium thermodynamics

(23) $$r=-\frac{L}{T}\Delta_{r}G$$

where *L* is a constant and *T* is temperature [Demirel, 2014]. Due to the minus sign, the more negative Δ_*r*_*G* the greater the growth rate. Growth ceases at equilibrium, when Δ_*r*_*G* becomes zero. Thus, Gibbs energy of growth is the driving force of growth of all chemotrophic organisms [Von Stockar, 2014].

If a virus is multiplying in a cell, then it must have a more negative Gibbs energy of growth (Δ_*r*_*G*) than the cell. Otherwise, the synthesis of cell components will dominate over new virion synthesis, and thus the virus would not be able to overtake the cell metabolism. Since a virus and its host cell use the same ribosomes, precursors and enzymes to synthesize their components, catalytic activity should not be responsible for the virus taking over the cell. Thus, if catalytic activity is not what governs the process, it must be thermodynamics. Indeed, in terms of Eq. (23), both the host cell and the virus are at the same temperature and use the same pathways, implying that *T* and *L* are the same for both. Thus, the only parameter that determines growth rate is Δ_*r*_*G*. So, if the reaction rate of virus growth is greater than the growth reaction rate of its host cell, we may conclude that this occurs only due to lower Δ_*r*_*G*.

Every chemical reaction, including growth reactions, is characterized by its Gibbs energy change, which depends on the chemical nature of reactants and products. However, reaction Gibbs energy is also influenced by conditions in the reaction mixture, in particular on temperature, reactant and product concentrations, and intermolecular forces between reaction participants. Therefore, a reaction Gibbs energy under standard conditions was defined as a reference point. Any deviations from the standard conditions are taken into account by corrections to the standard Gibbs energy. Thus, mathematically, reaction Gibbs energy is defined by the equation

(24) $$\Delta_{r}G=\Delta_{r}G^{0}+R_{g}T\;\ln\left(Q\right)$$

where Δ_*r*_*G⁰* is the standard Gibbs energy of growth, *R*_*g*_ the universal gas constant, while *Q* is the reaction quotient [Atkins and de Paula, 2014]. The reaction quotient is defined through concentrations, *C*, activity coefficients, *γ*, and stoichiometric coefficients, *ν*, of reactants and products [Atkins and de Paula, 2014]

(25) $$Q=\frac{\prod_{products}{\left(C\;\cdot \;\gamma \right)}^{\nu }}{\prod_{reactants}{\left(C\;\cdot \;\gamma \right)}^{\nu }}$$

The first term on the right-hand side of Eq. (24), Δ_*r*_*G⁰*, takes into account the chemical nature of reactants and products. The second term, containing *Q*, describes the influence of reactant and product concentrations, through *C*'s, as well as nonideal behavior due to interactions between molecules in reaction mixture, through *γ*′s. Thus, in essence, the *Q* term covers conditions in the reaction mixture, the influence of which on biological processes has been studied in detail by Meurer et al. [2016, 2017], Wangler et al. [2018] and Greinert et al. [2020a, 2020b].

The focus of this paper is comparison of driving forces of growth of viruses and their host cells. A virus and its host cell have different chemical compositions and thus their empirical formulas differ. Since empirical formulas represent newly formed live matter in growth reactions, the main product, growth reactions of viruses and their hosts must be different (Supplementary Information 3). Therefore, since different reactions are being compared, the main difference between their Gibbs energies will come from the difference in the chemical nature of reactants and products. In other words, the greatest difference will be between the Δ_*r*_*G⁰* values of the two reactions. Thus, in this work the Δ_*r*_*G* in Eq. (23) was approximated with Δ_*r*_*G⁰* values of viruses and their host cells.

Standard Gibbs energies of growth, given partly in Figure 1 and fully in Table 2, are negative for all human tissues, bacteria and viruses. This implies that synthesis of molecules necessary for growth and reparation of human tissues, bacterial cells and reproduction of virions is spontaneous. Notice that viruses have lower standard Gibbs energies of growth than human tissues and bacteria (Table 2).

Poliomyelitis is manifested in two forms, gastrointestinal and paralytic. The gastrointestinal form occurs more frequently than the paralytic form. Why? The poliovirus is characterized by lower standard Gibbs energy of growth than the intestine and the neurons (Table 2). Moreover, the difference between standard Gibbs energy of growth of poliovirus and intestine is greater than between poliovirus and neurons (Figure 1a) implying that poliovirus will more probably attack intestine and less probably attack neurons. So, according to Gibbs energy analysis, both forms are possible, but with different probability. This fact explains why the paralytic form of poliomyelitis is less probable than the gastrointestinal form. The reason is the difference in ratios of Gibbs energies of growth between virus and two different hosts.

The situation is similar with bacteriophages λ, N4, and T7, and their host E. coli. The reaction rate of bacteriophage growth is greater than growth reaction rate of E. coli due to lower Δ_*r*_*G⁰* (Figure 1b). Therefore, in all the studied cases, after a virus penetrates the cell, new virions will be formed at higher reaction rate than the host cell components. Hepatitis A and B viruses, adenoviruses, polyoma viruses and their host tissues support the explanation of the lytic cycle through Gibbs energy analysis (Figure 1c).

The examples discussed above indicate the importance of the ratio of Gibbs energies of viruses and their host cells. Growth reactions of viruses and their hosts occur at certain rates and are competing processes, since they use the same precursors. The ratio of growth rates, *R*, indicates the efficiency of growth. Growth rate, according to Eq. (23), depends on Gibbs energy of growth. Thus,

(26) $$R=\frac{r\left(virus\right)}{r\left(host\right)}=\frac{\Delta_{r}G^{0}\left(virus\right)}{\Delta_{r}G^{0}\left(host\right)}$$

**Figure 2:**
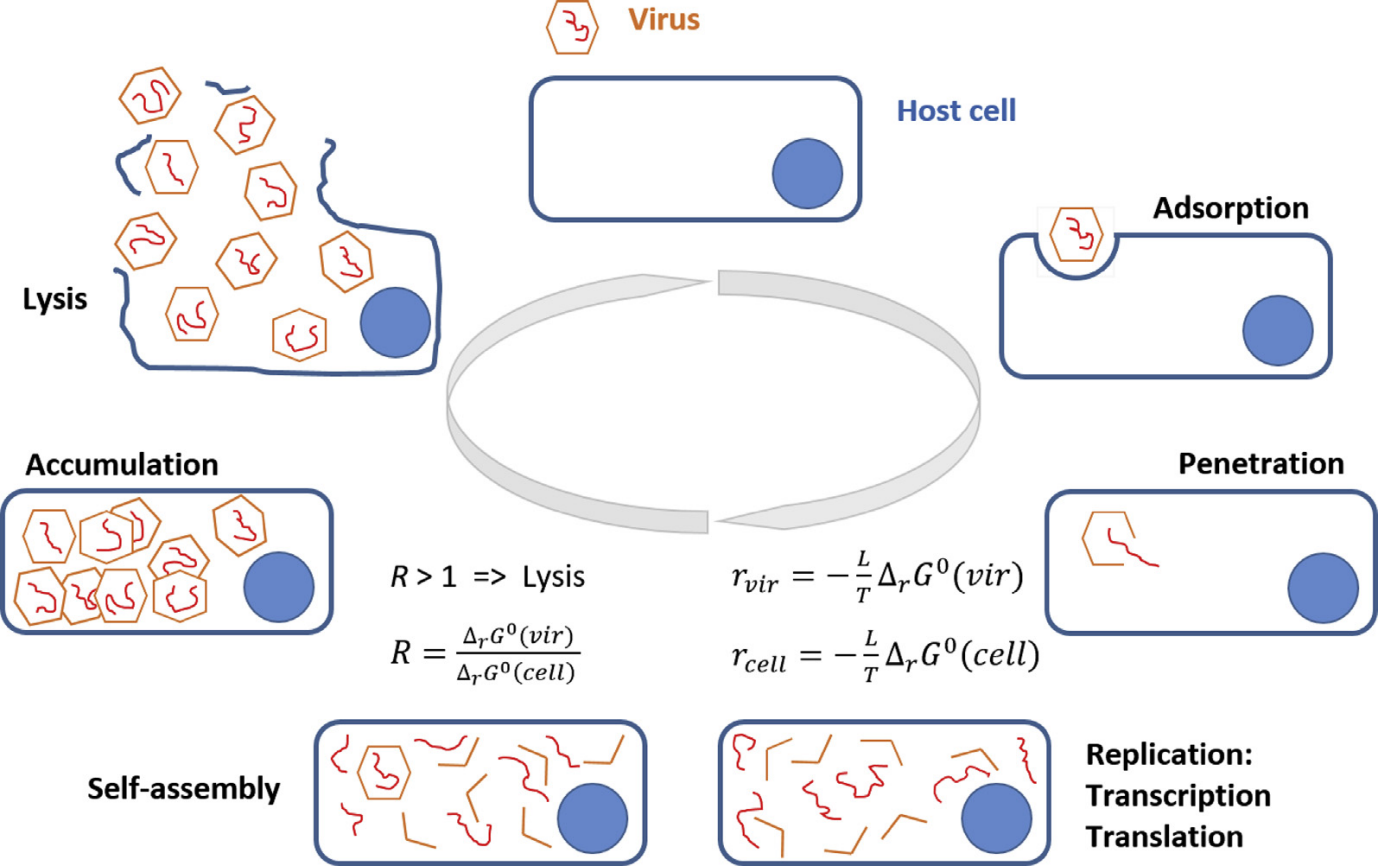
Viral lytic life cycle and its thermodynamic explanation.

After a virus penetrates a cell, there are two possible outcomes, depending on the ratio of standard Gibbs energies of growth of the virus and its host, *R*. If *R* is greater than one, formation of viral components is more favorable than that of the host cell components (Figure 2). Thus, viral components are synthesized faster than the host cell components. The host cell will accumulate virions and will burst, releasing the virions into the environment. This mechanism is employed by all viruses without a lipid envelope, since without lipids, they have a more negative Gibbs energy of growth than that of the host cell. On the other hand, if *R* is less than one, synthesis of viral components will be less favorable than those of the host cell. The virions will thus not be formed at a high enough rate to fill the entire cell, which will not burst due to being filled with virions. Instead the new virions will leave by budding. Thus, even though synthesis of new virions is not as favorable as synthesis of the host cell components, the fact that the product of virion synthesis is leaving the cell will lead to biosynthesis and formation of more product – synthesis of more virions. The growth reaction is shifted to the right. This mechanism is employed by viruses that have a lipid envelope, such as herpes and influenza (Table 2). The lipid envelope, due to its high Gibbs energy, makes the Gibbs energy of viral component synthesis less negative than that of the cell. So, the virions employ the other tactic to perform their life cycle.

There are two conditions for a virus to perform a lifecycle in its host:

1) The presence of a receptor on the host cell, for adsorption and penetration of the virus.
2) The Gibbs energy of growth of the virus must be lower than that of the host.

Thus, despite its lower Gibbs energy, a bacteriophage doesn't attack human cells, since they do not have an appropriate receptor. Furthermore, human herpes viruses (HHV1 and HHV2), after primary infection, remain latent in ganglions, performing the lysogenic life cycle, which is incompletely understood [Nicoll et al., 2012]. Virus-encoded latency-associated transcripts (LATs) suggests epigenetic regulation of the latent virus genome and the events that precipitate reactivation. The target tissues for HHV are skin epithelial cells and neurons, while the primary site of infection are the epithelial mucosal cells. HHV can perform two life cycles: lytic - causing clinical manifestations and lysogenic - latent. After primary infection in epithelial cells, the virus infects sensitive neurons and stays latent performing a lysogenic life cycle. Then, HHV can reactivate and be transported through axon back to the epithelium, causing clinical manifestations and performing a lytic life cycle. So, the same HHV can attack two different cell types, performing two different life cycles.

Neurons and epithelium have different chemical compositions and therefore differ in Gibbs energies of formation and Gibbs energies of growth. HHV with its lipid envelope is characterized by a Gibbs energy of growth of -13.64 kJ/C-mol, but without the lipid envelope its Δ_*r*_*G⁰* is -372 kJ/C-mol. Neurons are characterized by Δ_*r*_*G⁰* of -19.86 kJ/C-mol and epithelium by Δ_*r*_*G⁰* of -49.76 kJ/C-mol (Table 2).

The lytic and lysogenic cycles of HHV can be explained through Δ_*r*_*G⁰* analysis. Inside a cell, the virus loses its envelope. So, Δ_*r*_*G⁰* is -372 kJ/C-mol should be used as the characteristic parameter of HHV inside the epithelium. Epithelium is characterized by -49.76 kJ/C-mol. The difference of 322 kJ/C-mol enables the virus to have a higher growth rate and perform the lytic cycle in the epithelium. Later, HHV enters the dendrites of sensory ganglia and after retrograde transport to the nerve cell body, the virus encounters a “choice” of gene expression programs that determine the fate of the neuron: lytic or lysogenic [Nicoll et al., 2012].

The “choice of gene expression” implies that the virus makes a decision. However, viruses are chemicals by nature. Chemicals do not make any decisions but perform processes in accordance with the physical laws. Making decisions is a very complex information process that includes receptors, processors and effectors, which are not a part of any chemical, including viruses. So, it is more likely that a virus, due to its chemical nature, acts according to the physical laws. Thus, entering the sensitive neurons, a virion can retain its lipid envelope (Δ_*r*_*G⁰* = -13.64 kJ/C-mol) and remain latent, performing a lysogenic life cycle, since the difference in Δ_*r*_*G⁰* is only 6 kJ/C-mol, implying negligible difference in growth reaction rate (Eqn 23) between the virus and the neuron. Gibbs energy of growth determines the growth reaction rate. Thus, the similar values of Gibbs energy imply a similar growth rate and therefore simultaneous replication leading to the lysogenic cycle.

The alternative explanation is that after the virus penetrates the neuron, its DNA becomes a part of the nucleic acid subsystem of the neurons. Viral DNA can exist as a plasmid, but still represents a part of the subsystem DNA inside the neuron. Thus, the DNA subsystem has one common Δ_*r*_*G⁰* for both nucleic acids, resulting in the same reaction rate. The same growth reaction rate implies the lysogenic cycle. So, everything occurs in accordance with the thermodynamic and kinetic laws.

Moreover, HHV can perform the lytic cycle inside neurons causing encephalitis. A loss of the lipid envelope of HHV makes the difference in Δ_*r*_*G⁰* of virus and host cell larger, allowing very fast reaction rate, accumulation and lytic cycle inside neurons (viral Δ_*r*_*G⁰* without envelope is -372 kJ/C-mol, neuronal Δ_*r*_*G⁰* is -13.64 kJ/C-mol, so the difference is 360 kJ/C-mol allowing the lytic cycle).

Now we can conclude that HHV does not chose which life cycle it performs. It simply performs its life processes replication, biosynthesis, self-assembly and accumulation, at a rate depending on its characteristic Gibbs energy of growth. If there is a huge difference between a host cell and a virus, then the virus growth rate will be large, and it will accumulate and perform the lytic cycle. Notice that the difference between Gibbs energies of growth always exists between any host and virus, implying that even the lysogenic cycle appears (due to negligible difference). In a certain moment, the virus can use budding to leave its host causing reactivation of the virus from latent to active form.

**Figure 3:**
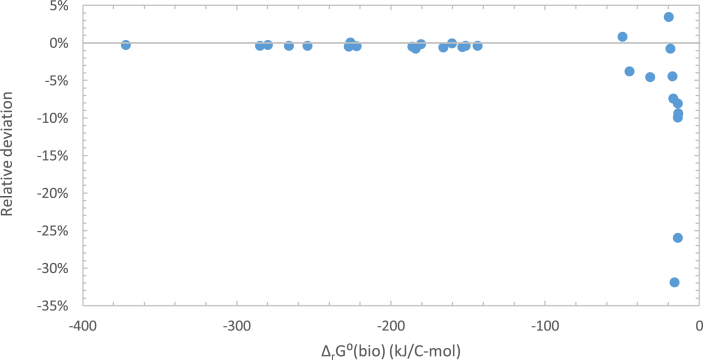
Comparison of Gibbs energies of growth calculated using the Battley equation, Δ_*r*_*G⁰*_*Battley*_ (*bio*), and the Roels equation, Δ_*r*_*G⁰*_*Roels*_ (*bio*). Relative deviation was calculated using the equation [Δ_*r*_*G⁰*_*Roels*_ (*bio*)- Δ_*r*_*G⁰*_*Battley*_ (*bio*)]/Δ_*r*_*G⁰*_*Battley*_ (*bio*).

The conclusions drawn above are independent of the method used to estimate thermodynamic properties of live matter. The results calculated using the Battley and Roels methods are presented in Figure 3 and Table 5. From Figure 3, it can be seen that in most cases the relative deviation doesn't exceed 10%. The only exceptions appear at low Gibbs energies of growth. This is because Gibbs energy of growth is calculated using Eq. (18) as the difference between products and reactants. In cases when large numbers are subtracted to give small results, even small differences become more pronounced. From Table 5, it can be seen that the same trend in Gibbs energies of viruses and their hosts can be observed, regardless of the method used for their estimation.

**Table 5:** Gibbs energies of growth calculated using the Battley, Δ_r_G⁰_Battley_, and Roels methods, Δ_r_G⁰_Roels_.

Name	Δ_r_G⁰_Battley_ (kJ/C-mol)	Δ_r_G⁰_Roels_ (kJ/C-mol)
Poliovirus	-186.14	-185.27
Gastrointestinal tract - small intestine (wall)	-16.85	-15.61
Brain-grey matter	-19.86	-20.56

Hepatovirus A	-184.07	-182.69
Hepatovirus B	-226.28	-226.47
Liver	-17.42	-16.65

Human herpes virus (entire virus)	-13.64	-12.37
Human herpes virus 1 (no envelope)	-371.99	-371.00
Epithelium	-49.76	-50.19
Neurons	-19.86	-20.56

Influenza	-13.95	-12.57
Adenovirus	-143.73	-143.20
Lung - parenchyma	-49.76	-50.19

BK polyomavirus (BKPyV) (Human polyomavirus 1)	-160.43	-160.37
Kidney	-13.72	-12.62

Flock house virus	-180.52	-180.27
Saccharomyces cerevisiae virus L-A	-153.56	-152.73
Saccharomyces cerevisiae virus L-BC	-151.61	-151.06
Saccharomyces cerevisiae	-15.90	-10.82

Enterobacteria phage T4	-227.32	-226.30
Enterobacteria phage N4	-254.10	-253.14
Enterobacteria phage T7	-279.87	-279.23
Enterobacteria phage lambda	-266.09	-265.11
Enterobacteria phage PRD1 (Bacteriophage PRD1)	-165.89	-164.91
Escherichia coli	-45.25	-43.55

Enterobacteria phage PRD1 (Bacteriophage PRD1)	-165.89	-164.91
Pseudomonas C12B	-18.67	-18.53

Bacillus phage phi29	-222.25	-221.30
Bacillus subtilis	-31.75	-30.32

Cyanophage Syn5 virus	-284.82	-283.76
Cyanobacteria Synechocystis PCC 6803	-13.74	-10.17

The influence of uncertainties in thermodynamic properties, discussed in Section 2.4, can be seen in Table 6. The last column in Table 6 contains numbers from uncertainty combinations that are the most unfavorable for the conclusions of this research. Gibbs energies of viruses were set to the least negative, while those of host cells were set to the most negative values allowed by the uncertainties. Even these data support the conclusions: the Gibbs energies of viruses that exit their host cell by lysis have a Gibbs energy of growth much more negative than their host cell.

**Table 6:** The influence of uncertainty on the conclusions of this research. The column “Worst-case Δ_r_G⁰” contains uncertainty combinations that are the most unfavorable for the conclusions of this research: the Gibbs energies of growth of viruses was increased by the error, making them less negative, while those of the host cells was decreased to make them more negative.

Name	Δ_r_G⁰ (kJ/C-mol)	Worst-case Δ_r_G⁰ (kJ/C-mol)
Poliovirus	-186.14 ± 30.76	-155.38
Gastrointestinal tract - small intestine (wall)	-16.85 ± 32.87	-49.72
Brain-grey matter	-19.86 ± 34.69	-54.56

Hepatovirus A	-184.07 ± 30.44	-153.63
Hepatovirus B	-226.28 ± 31.45	-194.82
Liver	-17.42 ± 32.25	-49.67

Human herpes virus (entire virus)	-13.64 ± 32.49	18.85
Human herpes virus 1 (no envelope)	-371.99 ± 29.97	-342.02
Epithelium	-49.76 ± 32.59	-17.17
Neurons	-19.86 ± 34.69	14.83

Influenza	-13.95 ± 32.29	18.34
Adenovirus	-143.73 ± 31.34	-112.40
Lung - parenchyma	-49.76 ± 32.59	-82.34

BK polyomavirus (BKPyV) (Human polyomavirus 1)	-160.43 ± 31.50	-128.93
Kidney	-13.72 ± 32.81	-46.53

Flock house virus	-180.52 ± 31.25	-149.27
Saccharomyces cerevisiae virus L-A	-153.56 ± 31.12	-122.45
Saccharomyces cerevisiae virus L-BC	-151.61 ± 31.25	-120.36
Saccharomyces cerevisiae	-15.90 ± 29.30	-45.20

Enterobacteria phage T4	-227.32 ± 30.68	-196.64
Enterobacteria phage N4	-254.10 ± 30.58	-223.52
Enterobacteria phage T7	-279.87 ± 30.66	-249.21
Enterobacteria phage lambda	-266.09 ± 30.53	-235.56
Enterobacteria phage PRD1 (Bacteriophage PRD1)	-165.89 ± 30.97	-134.92
Escherichia coli	-45.25 ± 30.84	-76.09

Enterobacteria phage PRD1 (Bacteriophage PRD1)	-165.89 ± 30.97	-134.92
Pseudomonas C12B	-18.67 ± 31.87	-50.54

Bacillus phage phi29	-222.25 ± 30.75	-191.50
Bacillus subtilis	-31.75 ± 31.30	-63.05

Cyanophage Syn5 virus	-284.82 ± 30.36	-254.46
Cyanobacteria Synechocystis PCC 6803	-13.74 ± 30.28	-44.01

## 4 Conclusions

In conclusion, all analyzed organisms have a negative standard Gibbs energy of formation and standard Gibbs energy of growth, which was also found to be an exothermic process. Furthermore, all analyzed viruses that exit their host cell by lysis have a lower Gibbs energy of component synthesis than their host cells. This difference makes synthesis of viral components more favorable than those of their host cells implying higher viral growth reaction rate. Thus, it seems that the Gibbs energy difference is the driving force the metabolism hijacking. On the other hand, all analyzed viruses that exit their host cell by budding have a Gibbs energy similar or lower than their host cells, due to the lipids in their viral envelopes. Therefore, these viruses employ another mechanism – new virions leave the host cell continuously as they are synthesized, making the synthesis reaction shift towards more product.

The results of this research imply that, if a virus performs a lytic cycle, its Gibbs energy of component synthesis is lower than that of its host cell. By changing the chemical composition of the virus, which implies change in Gibbs energy of component synthesis, it is possible to decrease the capability to attack the host cell (virulence). This can potentially open a strategically new approach to designing vaccines.

## Declarations

### Author contribution statement

Marko Popovic: Conceived and designed the experiments; Performed the experiments; Analyzed and interpreted the data; Wrote the paper.

Mirjana Minceva: Analyzed and interpreted the data; Wrote the paper.

### Funding statement

This research did not receive any specific grant from funding agencies in the public, commercial, or not-for-profit sectors.

### Competing interest statement

The authors declare no conflict of interest.

### Additional information

No additional information is available for this paper.

## Appendix A Supplementary data

Supplementary information 1 - Virus protein sequences Supplementary information 1 - Virus protein sequences

Supplementary information 2 - Virus DNA sequences Supplementary information 2 - Virus DNA sequences

Supplementary information 3 - Growth stoichiometry Supplementary information 3 - Growth stoichiometry
